# Left ventricular diastolic dysfunction in nonhuman primate model of dysmetabolism and diabetes

**DOI:** 10.1186/s12872-015-0133-y

**Published:** 2015-10-30

**Authors:** Haihua Gu, Yongqiang Liu, Shuang Mei, Bingdi Wang, Guofeng Sun, Xiaoli Wang, Yongfu Xiao, Michael Staup, Francine M. Gregoire, Keefe Chng, Yixin (Jim) Wang

**Affiliations:** Cardiovascular and metabolic diseases division, Crown Bioscience, Inc., 6 West Beijing road, Taicang, Jiangsu China; Crown Bioscience, Inc. at David H. Murdoch Research Institute, 150 N Research Campus drive, Kannapolis, NC USA

**Keywords:** Echocardiography, Left ventricular dysfunction, Diabetes, Ejection fraction

## Abstract

**Background:**

Diabetes is one of the major risk factors for cardiomyopathy and heart failure with reduced ejection fraction (EF) and highly associated with left ventricular (LV) dysfunction in human. This study aimed 1) to noninvasively assess cardiac function using echocardiography; 2) to test the hypothesis that like diabetic human, cardiac function may also be compromised; in spontaneously developed obese, dysmetabolic and diabetic nonhuman primates (NHPs).

**Methods:**

Cardiovascular functions were measured by noninvasive echocardiography in 28 control, 20 dysmetabolic/pre-diabetic and 41 diabetic cynomolgus monkeys based on fasting blood glucose and other metabolic status.

**Results:**

The LV end-systolic volume (ESV) was higher while end-diastolic volume (EDV, 12 ± 5.7 mL) and EF (63 ± 12.8 %) significantly lower in the diabetic compared to control (14 ± 7 mL and 68 ± 9.8 %) group, respectively. The E/A ratio of LV trans-mitral peak flow rate during early (E) over late (A) diastole was significantly lower in the diabetic (1.19 ± 0.45) than control (1.44 ± 0.48) group. E-wave deceleration time (E DT) was prolonged in the diabetic (89 ± 41 ms) compared to control (78 ± 26 ms) group. Left atrial (LA) maximal dimension (LADmax) was significantly greater in the diabetic (1.3 ± 0.17 cm) than control (1.1 ± 0.16 cm) group. Biochemical tests showed that total cholesterol and LDL were significant higher in the diabetic (167 ± 63 and 69 ± 37 mg/dL) than both pre-diabetic (113 ± 37 and 41 ± 23 mg/dL) and control (120 ± 28 and 41 ± 17 mg/dL) groups, respectively. Multivariable logistic regression analysis demonstrated that LV systolic (reduced EF) and diastolic (abnormal E/A ratio) dysfunctions are significantly correlated with aging and hyperglycemia. Histopathology examination of the necropsy heart revealed inflammatory infiltration, cardiomyocyte hypertrophy and fragmentation, indicating the myocardial ischemia and remodeling which is consistent with the LV dysfunction phenotype.

**Conclusions:**

Using noninvasive echocardiography, the present study demonstrated for the first time that dysmetabolic and diabetic NHPs are associated with LV systolic (increased ESV, decreased EF, etc.) and diastolic (decreased EDV and E/A ratio, prolonged E DT, etc.) dysfunctions, accompanied by LA hypertrophic remodeling (increased LADmax), the phenotypes similarly to those found in diabetic patients. Thus, spontaneously developed dysmetabolic and diabetic NHPs is a highly translatable model to human diseases not only in the pathogenic mechanisms but also can be used for testing novel therapies for cardiometabolic disorders.

## Background

Cardiovascular disease remains one of the leading causes of death in the United States, accounting 52 % type 2 diabetic (T2D) patients who have a higher risk of mortality at comparable levels of coronary artery disease to those without T2D [1–3]. On the other end, diabetes is a major risk factor for heart failure with preserved ejection fraction (EF), and is highly associated with left ventricular (LV) diastolic dysfunction in human [4–6]. A major limitation to understanding how diabetes exacerbates cardiovascular diseases has been the lack of good translational animal models with both diabetes and cardiovascular diseases. Although some rodent and rabbit models have been used to study the pathogenesis of diabetes, these models have not been very translatable to study the mechanisms that link metabolic and cardiovascular disorders [7]. Spontaneously developed obesity-associated T2D in the nonhuman primates (NHPs) during adulthood exhibits clinical features of obesity, insulin resistance, dyslipidemia, diabetes, and pancreatic pathology that are similar to those observed in humans [8–16]. Furthermore, its metabolic progression from insulin resistance through impaired glucose tolerance to overt diabetes, the pathological changes that occur in the pancreatic islets as diabetes develops, and the comorbidities that manifest as a consequence of disease progression are all comparable to human diseases.

Echocardiography has been widely used as an essential diagnostic tool in clinic to noninvasively examine cardiac structure, ventricular function, non-perfused myocardium, etc. because of convenient, rapid, economical, and none radiation [4, 17]. Among the echocardiography indices, EF and the E/A ratio of early to late trans-mitral Doppler inflow velocity (E/A) are commonly used to evaluate LV function in risk assessment of cardiac death. These studies showed that EF was a major determinant of long-term survival in patients with coronary artery disease [18]. Doppler images of the mitral valve annulus and trans-mitral propagation velocity have also been proposed to assess LV compliance and the severity of diastolic dysfunction [19, 20]. However, there have not been studies utilizing echocardiography to noninvasively characterize cardiac functions in NHPs with spontaneously developed dysmetabolism and T2D.

Therefore, the aims of this study are to test the hypothesis that like diabetic human, cardiac functions may also be compromised in spontaneously developed obese, dysmetabolic and diabetic NHPs, by 1) To establish an echocardiography method for noninvasive measurements of cardiac functions in NHPs; 2) to determine if these NHPs also have a compromised LV diastolic dysfunction; and 3) to examine the relationship between the LV diastolic dysfunction and the accompanying cardiovascular risk factors.

## Methods

Experiments were carried out in the entire colony of 89 cynomolgus monkeys of either genders then existed onsite, individually housed under ∼ 21 °C room temperature and a 12 h. light/dark (6 pm–6 am) cycle with continuous access to water ad libitum and controlled access to food with a complete nutritionally balanced diet (Shanghai Shilin Biotechnology, Inc., Shanghai, China) enriched with seasonal fruits and vegetables. These monkeys had no history of pharmaceutical treatment in the past 2 months whose pathophysiological conditions were stable.

The revised guidelines on diagnosis and classification of T2D in clinic from Diabetes Mellitus Expert Committee is fasting blood glucose (FBG) ≥126 mg/dL. There is currently no consensus in research community for the exactly criteria to define dysmetabolic and diabetic standard for NHP model. However, the FBG in normal young adult lean NHP population is typically ~20 mg/dL lower than that in healthy humans. Therefore, we pre-divided the 3 experimental groups using the below arbiter standard: control (*n* = 28, FBG < 85 mg/dL), pre-diabetic/dysmetabolic (*n* = 20, FBG = 85–125 mg/dL) and diabetic (*n* = 41, FBG > 125 mg/dL). The diagnosis of diabetes and dysmetabolism is not just based on a single FBG measurement, it is based on multiple measurements over the progressive history for at least 3 months, along with other metabolic parameters such as intravenous glucose tolerance test (ivGTT), body fat composition measured by dual-energy X-ray absorptiometry (DEX); HbA1c, insulin and lipids levels, etc.

### Experimental procedures

On the experimental day, monkeys were fasted overnight and received an intramuscular injection of ketamine (Fujian Gutian Pharmaceutical Co. Ltd., Fujian, China) at an initial dose of 10 mg/kg followed by maintenance doses of 5 mg/kg as needed. Body temperature was maintained at ∼ 37 °C by a thermostatically controlled warm water-circulating pad placed underneath the animal. Throughout the experimental period, animals were monitored on the vital signs which may trigger intervention (supportive and/or emergent care) and/or study termination including: oxygen saturations <90 %; body temperature <35 °C; heart rate (HR) <100 or >200 beats/min; respiratory rate < 20 or > 60/min, serious arrhythmia and respiratory artifacts, etc.

### Echocardiographic examination

Each monkey underwent a comprehensive transthoracic scanning with 2-dimensional, M-mode, and pulsed-wave Doppler echocardiography using an ultrasound system (ProSound SSD-3500SX, Hitachi Aloka Medical, Ltd. Tokyo, Japan). Qualified echocardiographic images were captured and stored for offline analysis with each parameter being taken from 3 to 5 consecutive cardiac cycles to obtain an average value. The LV inner dimensions at end-systolic (LVIDs) and end-diastolic (LVIDd), and septal dimension (LVSD) along with heart rate (HR) were measured using M-mode tracing from the parasternal long axis (PLAX) view. From these parameters, fractional shortening (FS), ejection fraction (EF), cardiac output (CO), end-diastolic (EDV), end-systolic (ESV) and stroke (SV) volumes were calculated. The LV diastolic filling function was assessed by trans-mitral flow velocities with pulsed-wave Doppler under the apical four-chamber view [21]. The peak trans-mitral inflow velocities at early (E) and late (A) diastole and E-wave deceleration time (E DT) were measured using pulsed-wave Doppler with the sample volume at the tip of mitral valve. Left atrial (LA) maximal dimension (LADmax) was measured by M-mode echocardiography from the parasternal long axis view. This is a prospective and single blind study with echocardiographic operators not knowing the animal metabolism condition when scanning animals and analyzing the data.

After completion of the echocardiographic scanning, animals were returned to their home cage and closely monitored by a trained personnel until completely recovery from anesthesia when the animals were in the upright position and food and water were then provided.

### Histopathological examination of the heart tissue

In 1 male and 1 female diabetic monkeys with LV diastolic dysfunction and LA remodeling who died naturally due to unknown reasons (aging, diabetes and its complications, etc.), necropsy was performed and the hearts were collected and placed in 4 % paraformaldehyde, and then imbedded with paraffin. Tissues were cut into 5 μm sections and stained with hematoxylin and eosin (H&E). Histopathological changes were examined via light microscopy by a histopathologist.

The experimental protocol and procedures were approved by the Institutional Animal Care and Use Committee (IACUC) of Crown Bioscience, Inc. and in accordance with the guidelines of Association for Assessment and Accreditation of Laboratory Animal Care (AAALAC).

### Statistical analysis

All the data were expressed as mean ± standard error (SE). Statistical analysis was conducted using SPSS 17.0 software (SPSS for Windows, version 21.0; IBM-SPSS, Chicago, Illinois, USA). Student *t*-test was used to compare the mean values between 2 experimental groups. One-way analysis of variance (ANOVA) was used for comparison of the mean values among 3 experimental groups.

Univariate logistic regression analysis was conducted to examine the relationships between the prevalence of LV dysfunction with other parameters such as age, fasting blood glucose, HbA1c, insulin treatment, gender, etc. To simplify the analysis, the below arbiter cut-off values were used. LV systolic dysfunction defined as EF <55 % and diastolic dysfunction as E/A ratio <1 or >2 were used to associate with aging (>10 years old), hyperglycemia (FBG > 85 mg/mL), HbA1c (>7 %), insulin treatment, gender, etc. Currently, there is no consensus in the research community for the exact definition of the above abnormality for NHP model. First, a multiple logistic regression analysis using a step-wise backward method was conducted with the prevalence of LV dysfunction as dependent and all other factors as independent variables, which were significantly associated with myopia prevalence in the univariate analysis. Multivariable logistic regression analysis with odds ratios and 95 % confidence intervals (CI) were then used to examine the associations between animals with certain risk factors and those without. Certain risk factors identified by univariate analyses were subjected to multivariable logistic regression analysis as independent variables. All *P*-values were 2-sided and considered statistically significant when < 0.05.

## Results

### Basic characteristics (Table 1)

**Table 1:** Animals Characteristics

	*Contr*ol	Pre-diabetes	Diabetes
Number, *n* (%)	28 (31.5 %)	20 (22.5 %)	41 (46.1 %)
Age (year)	11 ± 1	12 ± 2	18 ± 1^**##^
Weight (kg)	9 ± 1	9 ± 1	8 ± 1^*^
FBG (mg/dL)	66 ± 4	99 ± 3^**^	227 ± 20^**##^
HbA1c (%)	4.7 ± 0.6	5.2 ± 0.6	9.1 ± 0.8^**##^
CHO (mg/dL)	120 ± 7	113 ± 9	167 ± 15^**##^
TG (mg/dL)	63 ± 7	82 ± 8	217 ± 36^**##^
HDL (mg/dL)	54 ± 4	48 ± 4	48 ± 5^**^
LDL (mg/dL)	41 ± 4	41 ± 6	69 ± 9^**##^
CHO/HDL	2.2 ± 0.5	2.3 ± 0.1	3.5 ± 1.8^*#^

*& **P < 0.05 & 0.01 vs. Control, # & ##*P* < 0.05 & 0.01vs. Pre-diabetes

The diabetic group was older than the other 2 groups. The fasting blood glucose (FBG) was significantly higher in the pre-diabetic, and the highest in diabetic compared to control group. The blood lipid concentrations, including TG, CHO and LDL were significantly higher and HDL lower, thus, LDL/HDL ratio higher in the diabetic than control or pre-diabetic groups. There were no significant differences in age, body weight, HbA1c and blood lipid levels between the control and pre-diabetic NHPs.

### Cardiac functions measured by noninvasive echocardiography

There were no significant differences in LV dimensions (LVSd, LVIDs, LVIDd and LVPWd) and mitral valve parameters (MVAmax and VMVmax) among 3 experimental groups although the trans-mitral valve pressure gradient (MV ∆P) significantly elevated in the diabetic NHPs (Table 2).

**Table 2:** Echocardiography parameters

	Control (*n* = 28)	Pre-Diabetes (*n* = 20)	Diabetes (*n* = 41)
LV dimensions			
LVSd (mm)	3.7 ± 0.2	3.9 ± 0.1	3.4 ± 0.2
LVIDs (mm)	11.9 ± 0.1	11.6 ± 0.2	11.9 ± 0.1
LVIDd (mm)	21.4 ± 0.4	19.9 ± 0.4	19.3 ± 0.4
LVPWd (mm)	4.8 ± 0.1	5.0 ± 0.1	4.6 ± 0.4
Mitral Valve parameters			
MVAmax (cm^2^)	1.1 ± 0.1	1.1 ± 0.1	0.9 ± 0.1
VMVmax(cm/sec)	83 ± 4	79 ± 4	84 ± 4
MV ∆P (mmHg)	2.6 ± 0.3	2.6 ± 0.3	3.0 ± 0.4^*##^

*P < 0.05, vs. Control; ## *P* < 0.01, vs. Pre-diabetes

#### Left ventricular systolic dysfunction (Fig. 1)

**Fig. 1:**
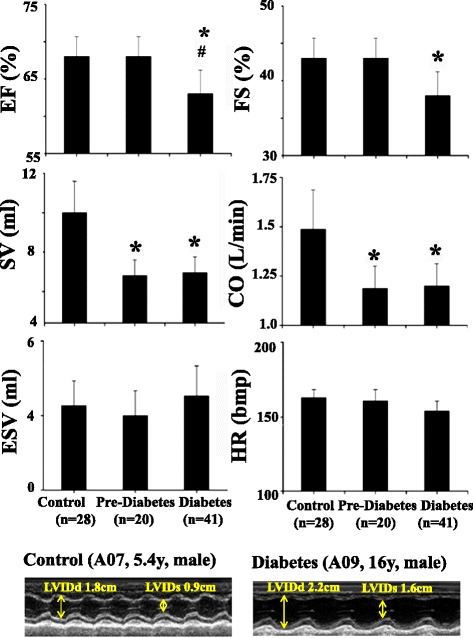
Left ventricular systolic dysfunction in control, pre-diabetic/dysmetabolic and diabetic NHPs. Group mean values +/− SE of LV ejection fraction (EF), stroke volume (SV), end-systolic volume (ESV), fractional shortening (FS), cardiac output (CO) and heart rate (HR). *P < 0.05 vs. control, # *P* < 0.05 vs. pre-diabetes. The original M mode echocardiography images in parasternal long axis for measurement of left ventricular inter dimensions at end-diastole (LVIDd) and end-systole (LVIDs) were obtained from a control and a diabetic monkey

The LV ejection fraction (EF), fraction shortening (FS), stroke volume (SV) and cardiac output (CO) were significantly lower in the diabetic compared to both control and pre-diabetic groups. Both the SV and CO, but not EF and FS were significantly lower in the pre-diabetic than control group. The LV end-systolic volume (ESV) and heart rate (HR) were not significantly different among 3 groups. The original M mode images obtained from the representative animals showed expended LV inter dimension at both end-diastolic (LVIDd) and end-systolic (LVIDs) phases in the diabetic compared to control one.

#### Left ventricular diastolic dysfunction (Fig. 2)

**Fig. 2:**
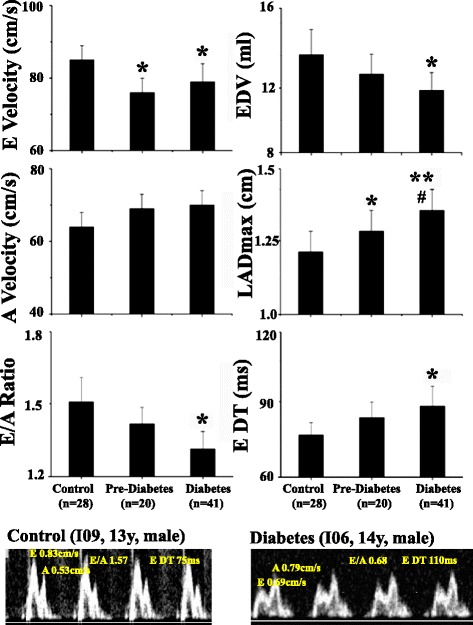
Left ventricular diastolic dysfunction in control, pre-diabetic/dysmetabolic and diabetic NHPs. Group mean values +/− SE of trans-mitral peak flow rate during the early (E-wave) and late (A-wave) diastole, E/A ratio, LV end-diastolic volume (EDV), left atrial maximal dimension (LADmax), LV E-wave deceleration time (E DT). *& **P < 0.05 & 0.01 vs. control, ## *P* < 0.01 vs. pre-diabetes. The original pulsed-wave Doppler echocardiography images showed mitral peak inflow velocities at early (E) and late (A) diastole and E wave deceleration time (E DT) measured with the sample volume at the tip of mitral valve from a control and a diabetic monkey

The peak mitral flow rate during early (E-wave) but not late (A-wave) diastole was significantly lower in both the pre-diabetic and diabetic compared to control group. As a result, E/A ratio was significantly lower only in the diabetic compared with control group. The original pulsed-wave echocardiographic images obtained from the representative animals showed that the E/A ratio was reversed in the diabetic compared to control animal. The LV end-diastolic volume (EDV) was significantly lower and E-wave deceleration time (E DT) prolonged only in the diabetic, but not pre-diabetic compared to control group. The maximum LA dimension (LADmax) was significantly higher in the pre-diabetic and the highest in diabetic compared to control group, indicating LA remodeling resulted from LV dysfunction.

#### Association of left ventricular dysfunctions with aging, hyperglycemia and insulin treatment (Fig. 3)

**Fig. 3:**
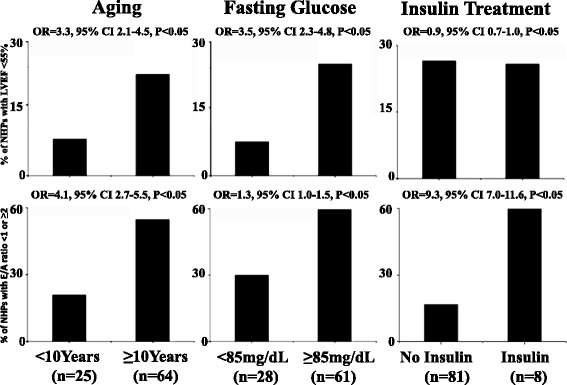
Association of left ventricular systolic (EF < 55 %) and diastolic (E/A < 1 or ≥ 2) dysfunction with aging (≥ 10 years old), hyperglycemia (FBG ≥ 85 mg/mL) and insulin treatment for more than 6 months

The incidences of low EF and abnormal E/A ratio in old and hyperglycemic NHPs were significantly higher than those in young (Left) and normal glycemic (Middle) ones. Consistently, in 32 NHPs with HbA1c >7 %, there were 30 (94 %) with low EF, while in 57 NHPs with HbA1c ≤7 %, there were only 18 (32 %) with low EF, the difference in the incidence of LV dysfunctions between the normal and high HbA1c group being statistically significant with OR = 32.5, CI 95 % 29.0–36.0, *P* < 0.05. The incidence of abnormal E/A ratio, but not low EF was significantly higher in overly diabetic NHPs with insulin treatment for more than 6 months than those without insulin treatment (Right). In contrast, the gender seemed not an independent predictor of LV dysfunctions (data not shown).

### Histopathology of cardiomyopathy (Fig. 4)

**Fig. 4:**
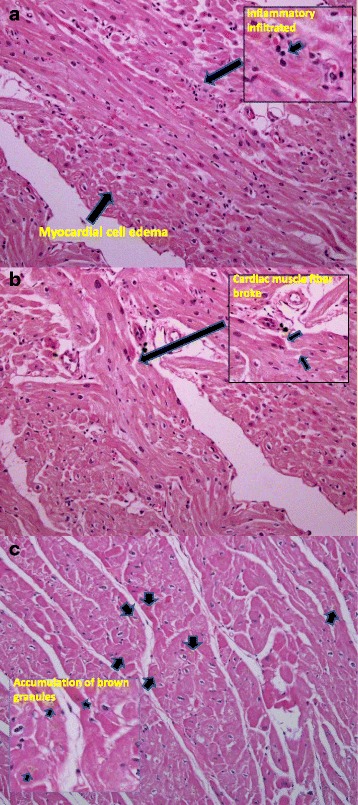
H&E staining from 2 representative diabetic monkeys with left ventricular diastolic dysfunction (Amplification: 20X for the original and 40X for corner image). a & b: A 23 year old female monkey with hyperglycemia (FBG: 429 mg/mL, HbA1c: 10.4 %), significantly reduced E/A (0.64) as well as increased E DT (144 ms) and LADmax (1.76 cm). **a** Focal myocardial inflammation: Inflammatory cell infiltration around the small vascular and light myocardial cell edema (indicated by ➡). **b** Broken myocardial muscle fibers with increased eosinophils (indicated by ➡). **c** A 21 years old male monkey with hyperglycemia (FBG: 247 mg/mL, HbA1c: 11.2 %), significantly reduced E/A (1.01) as well as increased E DT (159 ms) and LADmax (1.31 cm). Sings of myocardial ischemia and cardiac muscle atrophy: Accumulation of brown granules at the nuclear poles of the cardiac muscle fiber cytoplasm (indicated by ➡)

In 2 representative diabetic monkeys with significant LV diastolic dysfunction and LA remodeling revealed by echocardiography, histopathological examination of the necropsy heart was performed postmortem. In a 23 year old female animal with sever hyperglycemia and significantly reduced E/A ratio, there were focal myocardial inflammation with inflammatory cell infiltrated around the small vascular and myocardial edema (A), as well as some signs of myocardial ischemia such as of myocardial muscle fiber fragmentation and increased eosinophils (B). In another 21 year old male animal with diabetes and reduced E/A ratio, the H&E staining showed signs of myocardial ischemia and cardiac muscle atrophy with accumulation of brown granules at the nuclear poles of the cardiac muscle fiber cytoplasm (C).

## Discussion

Using noninvasive echocardiography, the present study detected left ventricular dysfunction for the first time in spontaneously developed obese, dysmetabolic and diabetic NHPs and its interaction between the LV dysfunctions and metabolic disorders, which is consistent with human disease.

The optimal performance of LV depends on its converting ability between systolic ejection and diastolic filling. Strong systolic contractility causes quick rise of LV pressure that enables blood to be effectively ejected into the aorta under high pressure gradient between the LV and aorta, while good diastolic compliance enables blood quickly and effectively to fill the LV from LA under low pressure condition that prompts the next contraction phase. Either systolic or diastolic dysfunction would compromise cardiac function to effectively pump blood for supplying the body needs.

### Systolic dysfunction

Rubler et al. first described the existence of ‘diabetic cardiomyopathy’ in 1972 based on some adult diabetic patients with chronic heart failure that could not be explained by coronary artery disease, hypertension, valvular heart disease, or alcoholism [22]. In prospective studies, approximately half of the heart failure patients have abnormal resting EF [23, 24], which is an important prognostic variable for survival prediction [8]. This hypothesis is supported by the data from Mayo clinic demonstrating a significantly worse survival in asymptomatic diabetic patients with EF <50 % [25]. Similar to the large epidemiological investigation in human [26-28], the present study demonstrated a significantly lower EF, along with FS, CO and SV in the diabetic compared with non-diabetic NHPs, indicating compromised LV contractility with global systolic dysfunction in this colony. The reference standard from American Society of Echocardiography sets resting EF <55 % as abnormal LV systolic function with 45–55 % as mild, 30–45 % as moderate, and <30 % as severe reduction [29]. According to this standard, the present data demonstrated that both aging and hyperglycemia strongly associated with the incidence of LV systolic dysfunction (low EF). Insulin treatment represents the advanced stage of diabetes progress, thus should also be associated with LV systolic dysfunction, however, it did not reach statistical significance in the present study, probably due to the weak statistical power resulted from small number of insulin treated animals included in this study.

Elevation of LV ESV also indicates reduced systolic function. Multivariate analysis identified ESV as an independent predictor of cardiac death after adjustment for perfusion and EF data. Tali et al. reported that if ESV = 70 mL is defined as a threshold, patients with severe perfusion abnormalities but ESV ≤ 70 mL had very low cardiac death rates (0.4 %/year), whereas patients only with mild/moderate perfusion defects but ESV > 70 mL had high cardiac death rates (8.2 %/year) [8]. In the present study there is no significant different in ESV among the 3 groups, which is probably due to LV systolic dysfunction in this colony of diabetic NHPs may not be severe enough to cause ESV reduction.

### Diastolic dysfunction

Diastole is the portion of the cardiac cycle that begins with aortic closure and ends with mitral closure. Increased LV stiffness or decreased LV compliance would cause abnormality in LV relaxation, alter the onset, rate, and extent of LV pressure decline, thus, building resistance to LV filling, which are important pathophysiologic mechanisms in LV diastolic dysfunction [30]. These changes create an abnormal LV pressure-volume relation so that higher filling pressure is required to maintain normal EDV and cardiac output. Mitral E-wave velocity primarily reflects early diastolic LA-LV pressure gradient that is also influenced by preload and LV relaxation function [31]. LV elastic recoil and diastolic pressure directly impact mitral E-wave velocity and time interval (e.g. E DT). Indeed, the present data demonstrated that dysmetabolic and diabetic NHPs are associated with decreased EDV and peak E-wave velocity as well as prolonged E DT. Mitral A-wave reflects late diastolic filling velocity as a result of LA contraction, which is not significantly different among the 3 groups. Thus, E/A ratio was significantly lower in diabetic NHPs. These results demonstrated LV diastolic dysfunction in dysmetabolic and diabetic NHPs, which is consistent with that in diabetic patients [20, 32, 33].

### Left atrial remodeling

LA remodeling is a continuous process under chronic diastolic burden or as long as cardiovascular risk factors or diseases still persist. Thus, LA enlargement becomes a reliable indicator of advanced diastolic dysfunction, regardless of LV systolic function, underlying cardiovascular disease or LV hypertrophic remodeling [34]. Patients with LV systolic dysfunction and heart failure showed that LA systolic and diastolic dimensions were independent predictors of exercise capacity and cardiovascular events (cardiac death or hospitalization for heart failure) [35]. In deed in the present study, diabetic NHPs presented with greater LADmax than controls, a strong evidence of LA remodeling, which could be a morphological adaptation to LV dysfunction or direct effects of hyperglycemia.

There is evidence supporting the existence of diabetes-induced direct structural and functional alterations in the myocardium [36]. Glycosylation products accumulate and cross-link with collagen in blood vessel walls and interstitial tissues contributing to micro-angiopathy. Cross-linking of proteins by glycosylation products also facilitates inflammatory cell invasion and LDL deposition in the vascular walls leading to atherosclerosis and focal inflammation, which in turn would cause myocardial ischemia. In diabetes, the equilibrium between energy production and utilization is disrupted in the myocardium [37]. Hyperglycemia results in increased intracellular glucose concentrations in myocardial cell and glucose metabolism products, which in turn increases the osmotic pressure in myocardium that stimulates the influx of water leading to cellular edema, thus, cardiomyocyte rupture. Oxidative imbalance-induced increase in oxidative stress, cardiomyocyte apoptosis and hypertrophy, as well as myocardial edema and fibrosis, have been suggested to be the key pathological features of development and progression of diabetic cardiac complications [38]. In the present study, the histopathological examination with the evidences of inflammatory infiltration in the myocardium as well as cardiomyocytes hypertrophy, edema and fragmentation is consistent with the echocardiographic observations on LV remodeling and LA diastolic dysfunction.

Diabetes has such an important influence on the development of congestive heart failure that it has been incorporated as a risk factor in the guidelines by American College of Cardiology/American Heart Association [39]. Hyperglycemia causes arterial basement membrane glycosylation that is a strong risk for atherosclerosis and associates with the development of diabetes-induced LV diastolic dysfunction [40-43]. Furthermore, high HbA1c was also an independent risk factor for LV dysfunction, e.g., each 1 % increase in HbA1c value has been associated with an 8 % increase in the risk of heart failure [42, 43]. Diabetes is often accompanied by dyslipidemia. Indeed the present data showed that the blood concentrations of triglyceride, total and LDL cholesterols were elevated and HDL reduced, thus, the ratio of LDL/HDL increased. It has been well-established that dyslipidemia closely associated with abnormalities in LV functions, which is also a high risk factor for atherosclerosis. Therefore, the accompanied dyslipidemia in the current NHP model of dysmetabolism and diabetes also contribute at least partially to the pathogenesis of cardiac dysfunction.

## Conclusions

Using noninvasive echocardiography, the present study demonstrated for the first time that obese, dysmetabolic and diabetic NHPs are associated with LV systolic (increased ESV, decreased EF, etc.) and diastolic (decreased EDV and E/A ratio, prolonged E DT, etc.) dysfunctions similarly to that in diabetic patients. In addition, similar pathogenic characteristics and accompanying risk factors are observed in both humans and NHPs. This makes the current NHP model of spontaneously developed obese, dysmetabolism and diabetes a unique translational tool not only for studying early development and environmental factors that affect the pathogenesis of obesity, dysmetabolism and diabetes, but also can be used for testing novel pharmacological interventions for cardiometabolic disorders.

## Abbreviations

A- or E-wave (cm/sec): Trans-mitral peak flow rate during the late or early diastolel
CHO (mg/dL): Total cholesterol
CO (L/min): Cardiac output
E DT (ms): E-wave deceleration time
EDV or ESV (mL): End-diastolic or end-systolic volume
EF (%): Ejection fraction
FBG (mg/mL): Fasting blood glucose
FS (%): Fractional shortening
HbA1c (%): Hemoglobin A1c
HDL or LDL (mg/dL): High or low density lipoprotein
LA or LV: Left atrial or ventricle
LADmax (cm): Left atrial maximal dimension
LVIDd or LVIDs (mm): Left ventricular inner dimension at end-diastole or end-systole
LVPWD (mm): Left ventricular posterior wall dimension
LVSD (mm): Left ventricular septal dimension
MV ∆P (mmHg): Trans-mitral valve pressure gradient
MVAmax (cm^2^): Maximal mitral valve area
MVVmax (cm/sec): Maximal trans-mitral valve velocity
NHP: Nonhuman primate
SV (mL): Stroke volume
T2D: Type 2 diabetes
TG (mg/dL): Triglyceride
